# *NQO1* C609T polymorphism and lung cancer susceptibility: Evidence from a comprehensive meta-analysis

**DOI:** 10.18632/oncotarget.21084

**Published:** 2017-09-19

**Authors:** Jiawen Huang, Huiran Lin, Xiaosong Wu, Weijun Jin, Zhidong Zhang

**Affiliations:** ^1^ Department of Pharmacy, The First Affiliated Hospital of Jinan University, Guangzhou 510630, Guangdong, China; ^2^ Animal Experimental Management Center, Public Technology Service Platform, Shenzhen Institutes of Advanced Technology, Chinese Academy of Sciences, Shenzhen 518055, Guangdong, China

**Keywords:** NQO1, C609T, polymorphism, lung cancer, meta-analysis

## Abstract

A variety of case-control studies have been performed to assess the correlation between *NQO1* C609T polymorphism and the risk of lung cancer, but an explicit consensus has not been reached. We conducted this updated meta-analysis to identify the function of *NQO1* C609T polymorphism in lung cancer risk. All relevant literature was retrieved from the PubMed, EMBASE, CNKI, and WanFang databases before April 2017. A total of 37 studies (29 articles) with 8493 cases and 10,999 controls were included. Odds ratios (ORs) and 95% confidence intervals (CIs) were used to assess the strength of relations. We found that the *NQO1* C609T polymorphism did not correlate with the risk of lung cancer in the overall analysis. In addition, no statistical significance was observed in the analysis stratified based on ethnicity, control source, quality score, or smoking status. A significant association was found in the subgroup of small cell lung cancer risk. Despite some limitations, this meta-analysis indicates that the *NQO1* C609T polymorphism may not be associated with lung cancer risk. However, more epidemiological studies of larger samples and more ethnicities are needed to confirm these results.

## INTRODUCTION

Worldwide, lung cancer is one of the most common cancers and the leading cause of cancer-related mortality among men, and the fourth leading cause of cancer morbidity and the second leading cause of cancer mortality among women [1, 2]. In China, lung cancer ranks first among the causes of cancer-related death [3]. Despite improvement in multimodal therapies, the 5-year survival rate for lung cancer is less than 10%. A major reason for this outcome is that a large proportion of lung cancer patients are diagnosed at advanced stages. The definitive mechanism of lung cancer is not fully understood. Evidence suggests that lung cancer is a multifactorial disease caused by genetic and environmental interactions [4–6].

NAD(P)H: quinone oxidoreductase (NQO1), formerly called DT-diaphorase, is a flavoenzyme associated with carcinogen metabolism [7]. As a two-electron reductase, the NQO1 protein can detoxify highly toxic quinones to less toxic hydroquinone analogues. NQO1 also acts as an antioxidant enzyme *in vivo*. By maintaining antioxidant forms of ubiquinone and reducing the endogenous quinones, NQO1 protects cells from oxidative damage [8]. Research has demonstrated that NQO1 can regulate tumor suppressor gene p53 activity, and thus influence cancer cell life [9]. The human *NQO1* gene, consisting of 6 exons and 5 introns, is located on chromosome 16q22. Numerous single nucleotide polymorphisms (SNPs) of the *NQO1* gene have been identified, but the most frequently studied SNP is *NQO1* C609T [10–12]. *NQO1* C609T (rs1800566, Pro187Ser) polymorphism is a C-to-T allele base-pair mutation in exon 6 (position 609) of the *NQO1* gene. Such a gene mutation changes the encoded protein from a proline to a serine at position 187. This Pro187Ser mutated protein shows a reduced quinine reductase activity when compared with the wild-type protein [13].

Epidemiological studies have assessed the correlation between the *NQO1* C609T polymorphism and lung cancer risk, but conflicting conclusions remain. In addition, several meta-analyses have been performed to reach a consensus but failed. To clarify the current uncertainty, we performed this comprehensive updated meta-analysis by incorporating all available literature.

## RESULTS

### Study characteristics

**Figure 1:**
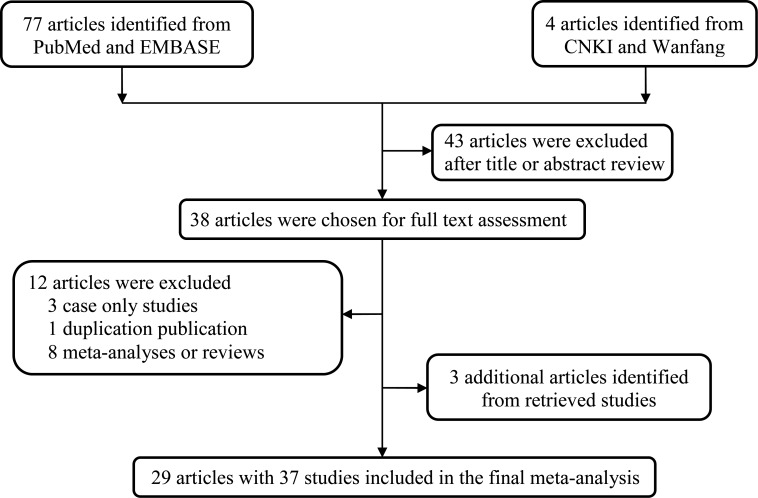
The main flowchart of this work

In the initial search stage, 81 potentially relevant published records were obtained. Through literature screening and abstracts reading, 38 articles were selected. We excluded 13 of those articles for the following reasons: 3 articles were case only research [14–16]; 1 article was duplicative [17], and 8 articles were meta-analyses [18–25]. However, 3 additional articles were identified from the references of the retrieval articles [26–28]. The workflow of the study selection process is shown in Figure 1. As a result, 37 studies (29 publications [21, 27–54]) with 8493 cases and 10,999 controls were used in the investigation (Table 1). Among the studies, 16 focused on Asians, 14 focused on Caucasians, 4 focused on African-Americans, 1 focused on Hawaiians, 1 focused on Hispanics, and 1 focused on a mixed population; 17 studies were hospital-based designs, 19 were population-based designs, and 1 was a mixed-based design. Of these studies, 23 were considered low quality (quality score ≤ 9), and 14 were considered high quality (quality score > 9). The control genotype frequencies in agreement with the Hardy-Weinberg equilibrium (HWE) were observed in 28 studies, but were not available in 9 studies.

**Table 1:** The baseline characteristics of all qualified studies in this meta-analysis

Name	Year	Country	Ethnicity	Control Source	Genotype method	Language	Case				Control				HWE	Score
							CC	CT	TT	All	CC	CT	TT	All		
Wiencke	1997	USA	Caucasian	PB	PCR	English	29	32		61	52	109		161	—	10
Wiencke	1997	USA	African-American	PB	PCR	English	77	39		116	83	53		136	—	10
Chen	1999	USA (Hawaii)	Asian	PB	PCR-RFLP	English	54	48	7	109	64	78	25	167	0.877	10
Chen	1999	USA (Hawaii)	Caucasian	PB	PCR-RFLP	English	81	49	5	135	105	62	4	171	0.137	10
Chen	1999	USA (Hawaii)	Hawaiian	PB	PCR-RFLP	English	61	18	4	83	60	39	3	102	0.258	10
Lin	2000	China	Asian	HB	PCR-RFLP	Chinese	12	63	20	95	41	73	22	136	0.268	3
Yin	2001	China	Asian	HB	PCR-CTPP	English	28	39	17	84	26	41	17	84	0.909	6
Xu	2001	USA	Caucasian	HB	PCR-RFLP	English	513	246	21	780	715	341	40	1096	0.933	6
Xu	2001	USA	Mixed	HB	PCR-RFLP	English	18	14	2	34	20	6	1	27	0.534	7
Lewis	2001	UK	Caucasian	HB	PCR	English	56	24	2	82	111	32	2	145	0.858	4
Benhamou	2001	France	Caucasian	HB	PCR-RFLP	English	85	55	10	150	105	62	5	172	0.243	10
Sunaga	2002	Japan	Asian	HB	PCR-RFLP	English	83	93	22	198	52	77	23	152	0.526	6
Hamajima	2002	Japan	Asian	HB	PCR-CTPP	English	87	71	34	192	240	286	114	640	0.076	6
Lin	2003	China	Asian	HB	PCR	English	57	141		198	95	237		332	—	9
Yin	2003	China	Asian	PB	PCR	Chinese	26	41	17	84	28	39	17	84	0.613	4
Lan	2004	China	Asian	PB	PCR	English	37	57	25	119	32	54	23	109	0.980	8
Liang	2004	China	Asian	HB	PCR-RFLP	Chinese	37	79	36	152	53	71	28	152	0.624	7
Alexandrie	2004	Sweden	Caucasian	HB	AS-PCR	English	345	168	11	524	368	153	9	530	0.124	7
Sorensen	2005	Denmark	Caucasian	PB	TaqMan	English	162	83	9	254	176	80	11	267	0.618	10
Saldivar	2005	USA	Caucasian	PB	PCR-RFLP	English	454	205	24	683	480	186	17	683	0.839	11
Saldivar	2005	USA	Hispanics	PB	PCR-RFLP	English	15	17	4	36	15	14	7	36	0.275	8
Saldivar	2005	USA	African-American	PB	PCR-RFLP	English	67	33	7	107	69	35	3	107	0.563	9
Skuladottir	2005	Denmark	Caucasians	Mixed	PCR-RFLP	English	108	45		153	227	119		346	—	6
Chan	2005	China	Asian	HB	PCR-RFLP	English	25	37	13	75	45	83	34	162	0.708	5
Bock	2005	USA	Caucasian	PB	TaqMan	English	93	37		130	87	57		144	—	7
Bock	2005	USA	African-American	PB	TaqMan	English	21	10		31	21	8		29	—	7
Lawson	2005	Finland	Caucasian	PB	PCR	English	244	109		353	243	117		360	—	10
Demirkan	2005	Turkey	Caucasian	PB	PCR	English	47	34	7	88	80	34	3	117	0.785	4
Yang	2007	Korea	Asian	HB	TaqMan	English	110	158	46	314	120	166	61	347	0.784	10
Eom	2009	Korea	Asian	HB	PCR-RFLP	English	122	265		387	148	239		387	—	9
Cote	2009	USA	Caucasian	PB	TaqMan	English	271	97	19	387	271	119	15	405	0.668	11
Cote	2009	USA	African-American	PB	TaqMan	English	77	32	4	113	79	36	6	121	0.478	10
Su	2009	China	Asian	PB	PCR-RFLP	Chinese	102	199	95	396	139	244	82	465	0.158	8
Timofeeva	2010	Germany	Caucasian	PB	MALDI-TOF MS	English	429	188		617	856	411		1267	-	10
Guo	2012	China	Asian	HB	PCR-LDR	English	187	327	168	682	171	282	144	597	0.192	8
Tian	2014	China	Asian	HB	PCR	English	88	171	132	391	215	307	141	663	0.109	10
Masroor	2015	India	Asian	HB	AS-PCR	English	45	48	7	100	71	26	3	100	0.743	6

HB, hospital based; PB, population based; PCR, polymerase chain reaction; AS-PCR, Allele specific-PCR; PCR-RFLP, PCR-restriction fragment length polymorphism; MALDI-TOF MS, Matrix-Assisted Laser Desorption Ionization-Time of Flight Mass Spectrometry; HWE, Hardy-Weinberg equilibrium.

### Meta-analysis results

**Figure 2:**
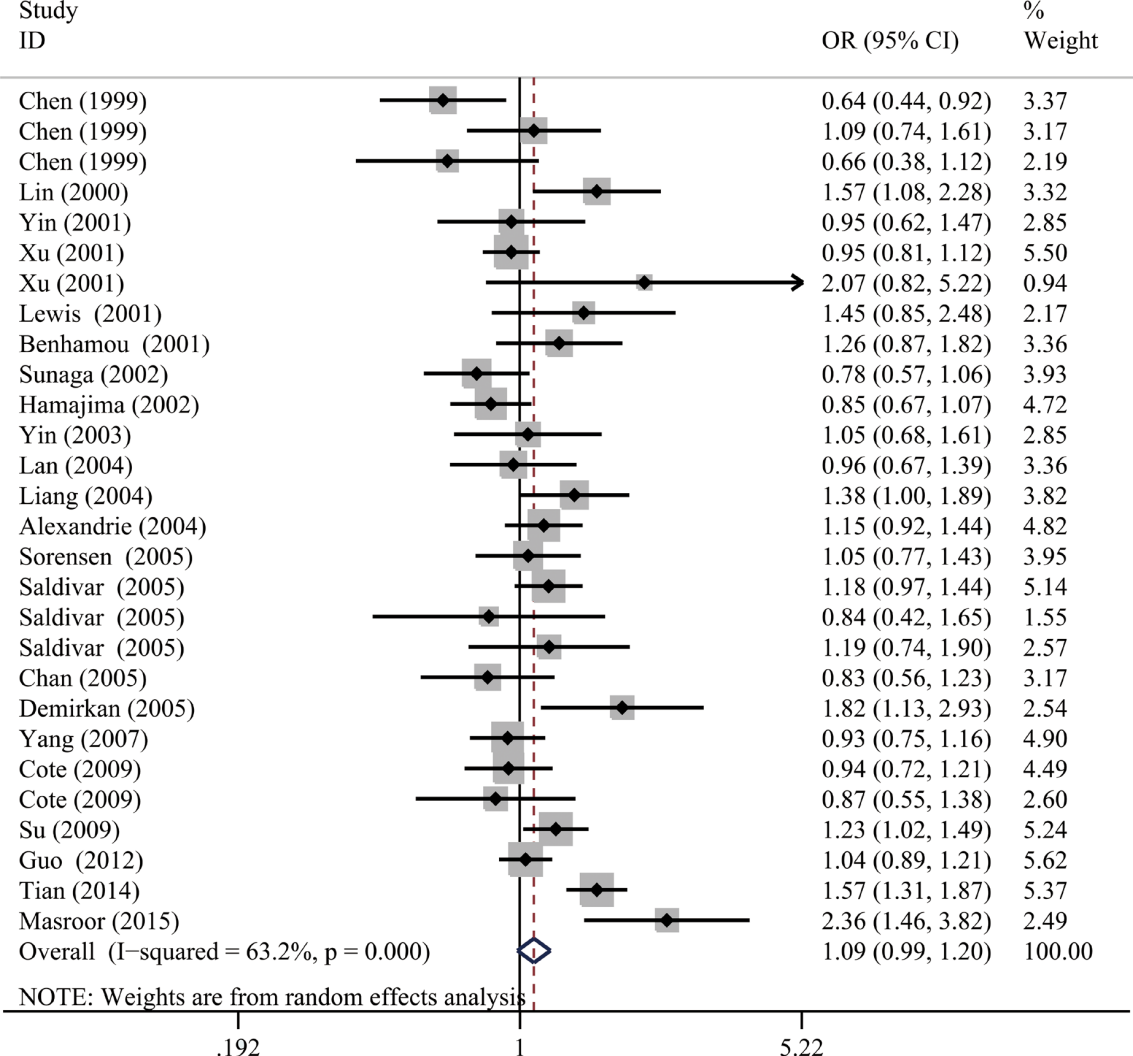
Forest plot for the correlation between the *NQO1* C609T polymorphism and lung cancer susceptibility under the allele comparison model. The horizontal lines represent the study-specific ORs and 95% CIs. The diamond represents the pooled results of OR and 95% CI.

The calculated results of the meta-analysis are shown in Table 2 and Figure 2. Overall, no significant correlation between the *NQO1* C609T polymorphism and lung cancer risk was observed in any of the genetic models (TT vs. CC: OR = 0.91, 95% CI = 0.74–1.12; CT vs. CC: OR = 1.08, 95% CI = 0.98–1.22; TT vs. CT+CC: OR = 1.13, 95% CI = 0.97–1.33; T vs. C: OR = 1.09, 95% CI = 0.99–1.20; and CT+TT vs. CC: OR = 1.04, 95% CI = 0.94–1.16).

**Table 2:** Meta-analysis of the correlation between NQO1 C609T polymorphism and lung cancer risk

Variables	No. of	Homozygous		Heterozygous		Recessive		Allele		No. of	Dominant	
	studies	TT vs. CC		CT vs. CC		TT vs. (CT+CC)		T vs. C		studies	(CT+TT) vs. CC	
		OR (95% CI)	*P*^het^	OR (95% CI)	*P*^het^	OR (95% CI)	*P*^het^	OR (95% CI)	*P*^het^		OR (95% CI)	*P*^het^
All	28	0.91 (0.74–1.12)	< 0.001	1.08 (0.96–1.21)	0.003	1.13 (0.97–1.33)	0.039	1.09 (0.99–1.20)	< 0.001	37	1.04 (0.94–1.16)	< 0.001
Ethnicity												
Asian	14	0.82 (0.62–1.09)	< 0.001	1.10 (0.91–1.34)	0.001	1.09 (0.88–1.34)	0.005	1.08 (0.93–1.26)	< 0.001	16	1.13 (0.94–1.36)	< 0.001
Caucasian	9	1.08 (0.77–1.51)	0.232	1.08 (0.97–1.20)	0.469	1.20 (0.90–1.59)	0.422	1.11 (0.99–1.24)	0.205	14	1.00 (0.89–1.13)	0.048
African	2	1.22 (0.36–4.15)	0.198	0.94 (0.63–1.41)	0.880	1.28 (0.38–4.29)	0.199	1.01 (0.73–1.41)	0.355	4	0.93 (0.69–1.25)	0.809
Hawaiian	1	1.33 (0.29–6.21)	—	0.45 (0.23–0.88)	—	1.67 (0.36–7.69)	—	0.66 (0.38–1.12)	—	1	0.52 (0.28–0.96)	—
Hispanics	1	0.57 (0.14–2.37)	—	1.21 (0.44–3.32)		0.52 (0.14–1.95)	—	0.84 (0.42–1.65)	—	1	1.00 (0.39–2.55)	—
Mixed	1	2.00 (0.17–23.9)	—	2.59 (0.82–8.18)	—	1.63 (0.14–18.9)	—	2.07 (0.82–5.22)	—	1	2.54 (0.85–7.58)	—
Source of control												
PB	13	1.05 (0.82–1.36)	0.320	1.00 (0.87–1.15)	0.274	1.16 (0.90–1.50)	0.247	1.03 (0.91–1.17)	0.043	19	0.95 (0.84–1.07)	0.067
HB	15	0.85 (0.74–1.12)	< 0.001	1.17 (0.98–1.38)	0.001	1.12 (0.91–1.38)	0.025	1.14 (0.99–1.32)	< 0.001	17	1.19 (1.01–1.40)	< 0.001
Quality score												
> 9	10	0.93 (0.71–1.22)	0.186	1.00 (0.86–1.17)	0.111	1.16 (0.81–1.67)	0.008	1.02 (0.85–1.22)	< 0.001	14	0.96 (0.83–1.11)	0.005
≤ 9	18	0.91 (0.74–1.12)	< 0.001	1.15 (0.98–1.36)	0.004	1.10 (0.97–1.33)	0.496	1.12 (1.00–1.20)	0.001	23	1.12 (0.97–1.30)	< 0.001
Smoking status												
Ever	6	1.11 (0.79–1.56)	0.113	1.09 (0.94–1.26)	0.511	1.09 (0.78–1.53)	0.225	1.08 (0.91–1.31)	0.088	14	1.04 (0.94–1.15)	0.283
Never	4	0.91 (0.48–1.74)	0.276	1.11 (0.79–1.57)	0.292	0.97 (0.53–1.76)	0.396	1.02 (0.69–1.51)	0.099	11	1.03 (0.82–1.28)	0.505
Lung cancer subtype												
Non-small cell	10	1.34 (0.90–1.99)	0.002	1.21 (0.99–1.48)	0.031	1.26 (0.94–1.71)	0.025	1.22 (1.00–1.48)	< 0.001	13	1.16 (0.96–1.40)	0.001
Small cell	3	2.57 (1.24–5.32)	0.730	1.24 (0.93–1.65)	0.481	2.38 (1.16–4.88)	0.813	1.35 (1.06–1.71)	0.358	3	1.33 (1.01–1.75)	0.388

Het, heterogeneity; HB, hospital based; PB, population based; NA, not available.

Further subgroup analysis by ethnicity, control source, quality score, and smoking status still did not yield a significant association, except for the heterozygous model and dominant model in the Hawaiian subgroup (CT vs. CC: OR = 0.45, 95% CI = 0.23–0.88; CT+TT vs. CC: OR = 0.52, 95% CI = 0.28–0.96) and the allele model in the quality score ≤ 9 subgroup (T vs. C: OR = 1.12, 95% CI = 1.00–1.20). In the subgroup analysis by lung cancer subtype, statistically significant increased risks were found among non-small cell lung cancer for T vs. C (OR = 1.22, 95% CI = 1.00–1.48) and small cell lung cancer for TT vs. CC (OR = 2.57, 95% CI = 1.24–5.32), TT vs. CT+CC (OR = 2.38, 95% CI = 1.16–4.88), T vs. C (OR = 1.35, 95% CI = 1.06–1.71) and CT+TT vs. CC (OR = 1.33, 95% CI = 1.01–1.75).

### Heterogeneity and sensitivity analysis

**Figure 3:**
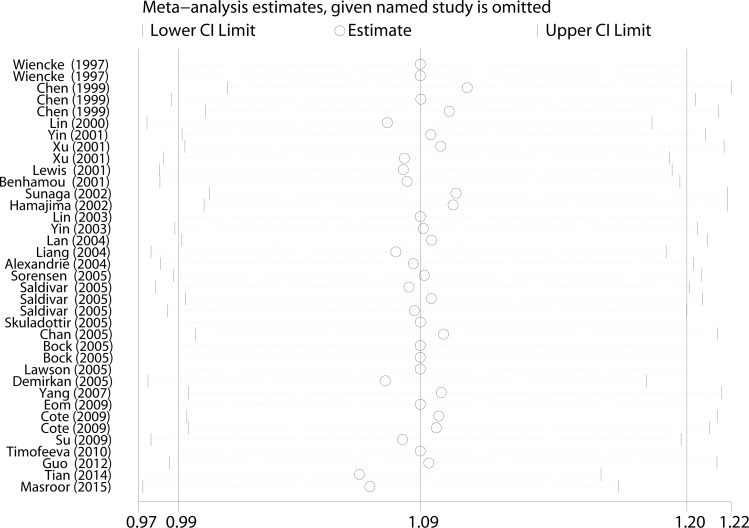
Sensitivity analysis of the association between *NQO1* C609T and lung cancer susceptibility under the allele comparison model Each point represents the recalculated OR after deleting a separate study.

Before calculating the ORs and 95% CIs, we used the *Q*-test and the I-squared statistics to test between-study heterogeneity. In the pooled analysis, significant heterogeneity exists among all five genetic models (*P* < 0.001, I^2^ = 55.2% for Homozygous, *P* = 0.003, I^2^ = 47.2% for Heterozygous, *P* = 0.039, I^2^ = 34.5% for Recessive, *P* < 0.001, I^2^ = 63.2% for Allele and *P* < 0.001, I^2^ = 57.0% for Dominant genetic models). Thus, the random-effect model was used to generate wider CIs. Moreover, we adopted a sequential leave-one-out sensitivity analysis to assess the influence of a single study on the combined ORs. After omitting each study, no substantial changes in ORs were observed, which suggested the robust and reliability of this meta-analysis (Figure 3).

### Publication bias

**Figure 4:**
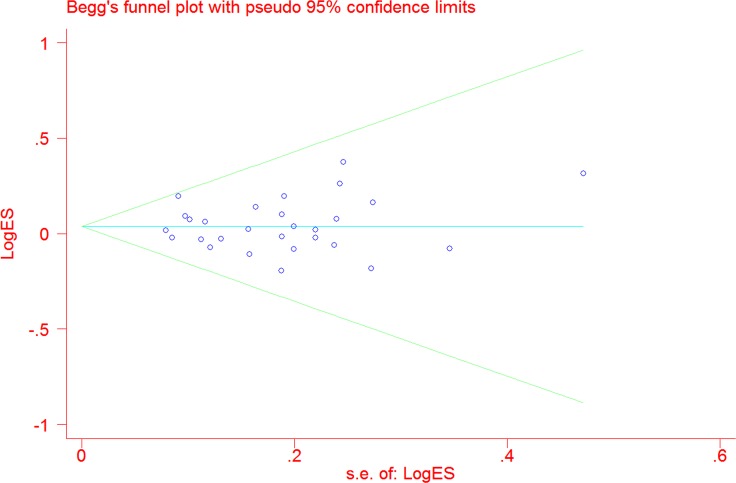
Funnel plot analysis to detect publication bias for *N*QO1 C609T polymorphism under the allele comparison model Each point represents a separate study.

No evidence of obvious asymmetry in Begg's funnel plots was identified by visual observation (Figure 4). In addition, Egger's test also indicated that no publication bias was shown among the studies (TT vs. CC: *P* = 0.48; CT vs. CC: *P* = 0.45; TT vs. CT+CC: *P* = 0.76; T vs. C: *P* = 0.93; and CT+TT vs. CC: *P* = 0.79).

### Trial sequential analysis

**Figure 5:**
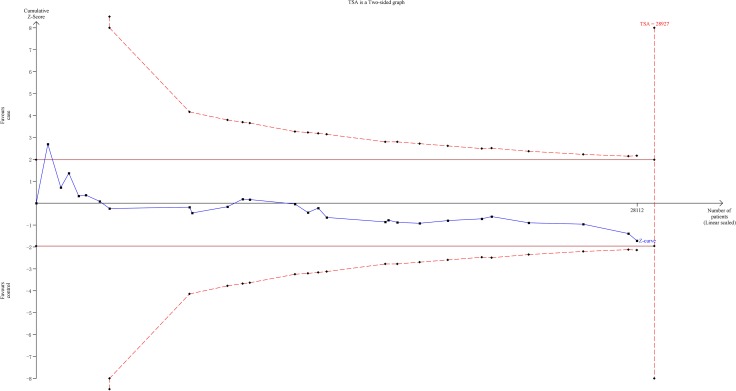
The required information size to demonstrate the correlation between *NQO1* C609T polymorphism with lung cancer susceptibility The solid blue line is the cumulative Z-curve. The dashed inward-sloping line to the left represents the trial sequential monitoring boundaries.

The TSA showed that the cumulative Z-curve failed to cross the trial monitoring boundary before reaching the required information size, suggesting that more trials are needed to further verify the conclusions (Figure 5).

## DISCUSSION

In this meta-analysis, we investigated the role of *NQO1* C609T polymorphism in lung cancer susceptibility. The obtained results suggest that no significant relation exists. To date, this meta-analysis includes the largest samples used in the investigation of the function of *NQO1* C609T in lung cancer susceptibility.

Numerous studies have investigated the activity of the *NQO1* gene C609T polymorphism in lung cancer risk. In 1999, Chen et al. [30] claimed that *NQO1* C609T polymorphism correlates with decreased lung cancer risk in Japanese. In another study conducted in England that consists of 82 lung cancer patients and 145 controls, Lewis et al. [31] failed to detect any correlation between *NQO1* C609T and lung cancer risk. However, Masroor et al. [32] reported that the *NQO1* 609TT genotype could increase the risk of lung cancer in an Indian population of 100 lung cancer cases and 100 healthy controls. Several meta-analyses have been performed to obtain a clear correlation between *NQO1* C609T and lung cancer risk. In a pilot meta-analysis conducted by Kiyohara et al. [22], only 10 studies were included, consisting of 2746 cases and 3902 controls. They found that the Ser allele is a protective factor against lung cancer in Asians, but not in Caucasians. In a meta-analysis that included 25 articles (32 studies) with 7522 cases and 9291 controls, Lou et al. [25] failed to observe any correlation between *NQO1* C609T polymorphism and lung cancer risk overall in African-Americans, East Asians, or Hispanics. However, the results suggested that *NQO1* C609T polymorphism might correlate with lung cancer risk in Caucasians.

This updated and comprehensive meta-analysis was performed to better elucidate the correlation between *NQO1* C609T polymorphism and lung cancer risk. However, we failed to observe any significant relation between *NQO1* C609T and lung cancer risk in the pooled analysis in any of the five genetic models. Subgroup analysis by ethnicity suggested that the T allele might be a protective factor in Hawaiians (CT vs. CC: OR = 0.45, 95% CI = 0.23–0.88; CT+TT vs. CC: OR = 0.52, 95% CI = 0.28–0.96). In this Hawaiian subgroup, only one study, with 83 cases and 102 controls was included, which limits the strength of this result. Thus, more case-control studies in Hawaiians are needed to elucidate the role of C609T polymorphism in lung cancer risk. In another subgroup stratified by quality score, we did not detect any correlation between *NQO1* C609T and lung cancer risk, yet the T allele acts as a risk factor for lung cancer in low-quality studies under the allele model (T vs. C: OR = 1.12, 95% CI = 1.00–1.20). The insufficient statistical power of the relatively low-quality studies should be also considered. It is possible that such insufficient statistical power might result in false positive results. When we stratified the analysis on the basis of lung cancer subtype, an increased risk of the *NQO1* C609T polymorphism on small cell lung cancer was observed. Previous pooled analyses have demonstrated that small cell lung cancer was more frequently observed in patients exposed to tobacco smoke [29]. The *NQO1* C609T polymorphism might result in failure of NQO1 protein to detoxify highly toxic quinones, and thus have some consequences for smoking-related small cell lung cancer risk.

In this meta-analysis, we adopted many measurements to be certain of the credibility of our conclusion. First, we incorporated all eligible studies not only written in English but also written in Chinese to expand the included sample numbers. Then the sensitivity analysis and the publication bias were assessed according to the Cochrane protocol. The sensitivity analysis indicated that the results were robust, and Funnel plots suggested that no obvious publication bias was observed. The TSA was also used to test the conclusion reliability of this meta-analysis. The Z-curve failed to cross the trial monitoring boundary before reaching the required information size. Thus, more studies are needed to confirm or refute this finding. However, the limitations must be pointed out when the results of this meta-analysis are interpreted. First, we found that most of the comparisons show significant between-study heterogeneity, which might reduce the validity of the conclusion. Second, the strength of correlation was obtained by use of unadjusted estimates. Adjustment analysis was absent because of the lack of original data, such as environmental factors, age, drinking status, and gene-environment interactions, which restrains further analysis for risk factors. Nearly all the included case-control studies were conducted among Asians and Caucasians. Other ethnicities, such as Africans, were not studied. Additional studies are needed to confirm such conclusions from other ethnicities, especially Africans. Publication bias and language bias still exist, because only published studies and only the studies written in English or Chinese are included.

The current meta-analysis provides evidence that *NQO1* C609T polymorphism is not correlated with lung cancer risk, from the perspective of the former case-control studies. Further high-quality investigations with more detailed environmental exposure information and larger sample sizes are warranted to confirm our findings.

## MATERIALS AND METHODS

### Publication search

We performed a comprehensive literature search of PubMed and EMBASE by adopting the combination of the following phrases: “polymorphism or single nucleotide polymorphism or SNP or variant” and “*NQO1* or quinone reductase or quinone oxidoreductase or DT-diaphorase or DTD,” and “lung cancer or lung neoplasm or lung carcinoma.” To enlarge the included studies, we also searched China National Knowledge Infrastructure (CNKI) and Wanfang databases using the above phrases in Chinese. In addition, eligible studies in the references of retrieved articles were also screened. The literature search was performed before April 2017 without any language publication restrictions. If an article contained two or more ethnic subpopulations, they were treated as separate studies. Only the largest study was included if two or more articles contained overlapping data. The designation and writing of this meta-analysis were under the guidelines of Preferred Reporting Items for Systematic Reviews and Meta-analyses.

### Eligibility criteria

All the included articles in this meta-analysis met the following criteria: (1) contained unrelated case-control studies, (2) evaluated the correlation of *NQO1* C609T polymorphism with lung cancer risk, (3) contained enough genotype distribution information to calculate ORs and 95% CIs, and (4) *NQO1* C609T genotype frequency in control subjects were in agreement of the Hardy-Weinberg equilibrium (HWE).

### Data extraction

Authors Jiawen Huang and Huiran Lin screened the articles and extracted data from all eligible studies, respectively. The data include first author's surname, publication year, country, ethnicity, the source of control subjects, genotyping methods, quality score, and numbers of cases and controls with CC, CT, and TT genotypes. Conflicting data were resolved by discussion after consensus was reached

### Quality assessment

The quality of each study was assessed by use of the quality assessment criteria [30–32]. The evaluation items were as follows: representativeness of case, representativeness of control, ascertainment of cancer, control selection, genotyping examination, HWE, and total sample size. Each study was evaluated on a scale from 0–15. Quality score of studies ranges from 0 to 15 points. Studies with scores ≤ 9 were of low quality, whereas those with scores > 9 were of high quality. The detail of score of quality assessment was listed in Supplementary Table 1.

### Statistical methods

All five genetic models, homozygous model (TT vs. CC), heterozygous model (CT vs. CC), recessive model (TT vs. CT + CC), dominant model (CT + TT vs. CC), and allele comparison (T vs. C) were adopted to investigate the correlation between *NQO1* C609T polymorphism and lung cancer risk. The strength of such correlation was assessed by calculating ORs with the corresponding 95% CIs. Stratification analyses were also performed by ethnicity, and source of control subjects, quality score, HWE in control subjects, smoking status, and lung cancer subtype. Between-study heterogeneity was analyzed by chi squared-based *Q*-test. When the studies were found to be homogeneous (with *P* > 0.10 for the *Q*-test), the fixed-effects model (the Mantel-Haenszel method) was used to estimate the pooled OR. Otherwise, the random-effects model (the DerSimonian and Laird method) was adopted. Sensitivity analysis was done by individually removing studies one by one and reanalyzing the pooled risk estimates. Begg's funnel plot and Egger's linear regression were used to estimate potential publication bias, with that asymmetric plot and a *P*-value < 0.05, indicating the presence of publication bias. HWE in the control subjects was tested by use of goodness-of-fit chi-squared test. A value of *P* < 0.05 was considered as departure from HWE. All statistical analyses were completed by STATA Version 11.0 software (Stata Corporation, College Station, TX). All the statistics were two-sided, with significant findings set at a *P*-value of less than 0.05.

### Trial sequential analysis

To avoid the random errors caused by repeated significance testing and dispersed data, we performed the trial sequential analysis (TSA). First, the required information size was evaluated by considering an overall type I error (a) of 5% and type II error (b) of 20%. Then TSA monitoring boundaries were constructed on the basis of required information size as well as risk for type I and type II errors. If the cumulative Z-curve (blue line) crosses a TSA monitoring boundary (red lines) before reaching the required information size, the robustness of evidence might be confirmed and no further trials are necessary. Otherwise, to get a robust conclusion, more trials are needed.

## SUPPLEMENTARY MATERIALS TABLE


