# Supplementary material for: NQO1 C609T polymorphism and lung cancer susceptibility: Evidence from a comprehensive meta-analysis
Source: Oncotarget. 2017 Sep 19;8(60):102301–9. doi: 10.18632/oncotarget.21084 (PMC5731955; doi:10.18632/oncotarget.21084)
Supplement: Supplementary file 1 [file oncotarget-08-102301-s001.pdf]

NQO1 C609T polymorphism and lung cancer susceptibility: Evidence from a comprehensive meta-analysis

SUPPLEMENTARY MATERIALS

Supplementary Table 1: Score of quality assessment

Criteria	Score
Representativeness of case	
Selected from population cancer registry	2
Selected from hospital	1
No method of selection described	0
Representativeness of control	
Population-based	3
Blood donors	2
Hospital-based	1
Not described	0
Ascertainment of cancer cases	
Histopathologic confirmation	2
Patient medical record	1
Not described	0
Control selection	
Controls matched with cases by age and sex	2
Controls matched with cases only by age or by sex	1
Not matched or not descried	0
Genotyping examination	
Genotyping done blindly and quality control	2
Only genotyping done blindly or quality control	1
Unblinded and without quality control	0
HWE	
HWE in the control group	1
HWE in the control group or not mentioned	0
Total sample size	
> 1000	3
501 - 1000	2
201 - 500	1
≤ 200	0
